# Sex differences in cognitive trajectories and practice effects in a cohort of older Londoners: The role of risk factors

**DOI:** 10.1177/13872877251339833

**Published:** 2025-05-07

**Authors:** Sima Toopchiani, Shireen Sindi, Neil Poulter, Sujin Kang, Chi Udeh-Momoh, Geraint Price, Miia Kivipelto, Lefkos Middleton, Oliver Robinson

**Affiliations:** 1 Ageing Epidemiology (AGE) Research Unit, School of Public Health, Imperial College London, London, UK; 2 Division of Clinical Geriatrics, Center for Alzheimer Research, Karolinska Institutet and Karolinska University Hospital, Stockholm, Sweden; 3 Imperial Clinical Trials Unit, School of Public Health, Imperial College London, London, UK; 4 Department of Epidemiology and Biostatistics, MRC Centre for Environment and Health, School of Public Health, Imperial College London, London, UK

**Keywords:** Alzheimer's disease, cognition, gender, risk factors, practice effects, sex differences

## Abstract

**Background:**

Sex differences in cognitive abilities have been reported; however, the underlying reasons remain unclear.

**Objective:**

To (i) investigate sex differences in cognitive performance, (ii) evaluate the contributions of established dementia risk factors to these differences, and (iii) examine the role of non-modifiable risk factors on sex differences in cognitive performance.

**Methods:**

Among 964 cognitively unimpaired participants (aged 60–85) of the UK CHARIOT-PRO Main Study, we assessed cross-sectional and longitudinal associations, over up to 3 years of follow-up, between sex and cognitive performance, using the Repeatable Battery for the Assessment of Neuropsychological Status (RBANS).

**Results:**

Sex differences, mostly favoring women were observed at baseline across almost all RBANS indices including the total scale (Cohen's d = 0.3, adjusted mean difference in score = −5.4, p < 0.001). Sex differences were observed in Practice effects (PEs), with men showing less PE in almost all cognitive domains including the total scale (adjusted 1.3, p = 0.002). Greater sex differences in PEs, were documented among the ‘older’ participants in two out of five cognitive domains including the immediate memory index (mean difference: older (69–85 years) group = −3.2, p = 0.002); younger (60–68 years) group = −0.8, p = 0.4). Sex differences were more pronounced among ‘Apolipoprotein-Ꜫ4 -carriers’ in three out of five domains including the total scale (mean difference in carriers = −2.6, p = 0.002); non-carriers = −0.7, p = 0.3).

**Conclusions:**

Sex differences in cognition and PE were observed after adjusting for risk factors associated with Alzheimer's disease. Future studies should also consider the effects of sex on non-modifiable risk factors and PEs to identify potential ‘masked’ neuropathology.

## Introduction

In most parts of the world, two-thirds of dementia cases are women.^1–6^ This discrepancy is partly explained by their greater longevity. However, studies investigating the risk of developing Alzheimer's disease (AD) have reported higher incidence in women even when compared to age-matched men.^7–9^ Despite the greater age-adjusted global prevalence of dementia among women, there is evidence to suggest that women possess an advantage in specific cognitive domains including verbal memory and tasks related to speed processing.^10^ This female advantage may reflect their greater resilience to age-related cognitive decline^11^ as well as differences in brain structure and function.^12^ Cognitive impairment, accompanied by signs of cognitive decline is a key hallmark in the clinical diagnosis of dementia. Cognition has been assessed through the administration of neuropsychological tests which consist of a comprehensive battery to assess various cognitive domains. Therefore, the accurate assessment of cognition is imperative for commencement of effective therapies and disease-modifying interventions.

Sex-specific normative values have been developed to mitigate potential biases in cognitive tests resulting from sex differences.^13^ Despite these efforts, the female advantage in cognitive tests persists and may contribute to the underestimation and masking of early cognitive impairment in women. Timely diagnosis of dementia is critical for the consideration of earlier interventions, delaying disease progression and improving the patients’ quality of life*.*

One of the key factors to consider is the impact of practice effects (PEs) on cognitive assessments among the sexes throughout the lifespan. PEs refer to improvements in cognitive performance due to repeated exposure to cognitive testing.^14^ PEs among ‘cognitively’ healthy individuals can potentially mask early signs of cognitive decline, hindering the diagnosis and treatment of dementia. Some studies have viewed PEs as a source of bias or error when handling and analyzing repeated measures of cognitive assessments.^15,16^ Others have suggested using PEs as a classifier in clinical trials^17,18^ to categorize or differentiate between groups of people and cognitive conditions. Conversely, a recent study found that PEs in persons with AD or mild cognitive impairment (MCI) may affect cognitive progression and sample size estimation (not considering PEs may lead to inaccurate sample size estimates).^19^ Studies investigating the effect of sex on PEs in cognitive assessments have been limited; however, in a recent study PEs were more pronounced among women.^20^ This finding suggests a potential role of sex in PEs and may be a significant variable contributing to why women often exhibit sustained performance in cognitive tests and may delay the diagnosis of cognitive impairment.

The Lancet Commission identified modifiable risk factors associated with and linked to increased dementia risk.^21,22^ However, epidemiological studies have reported persistent sex differences in dementia risk, even after adjusting for socioeconomic, lifestyle, and vascular risk factors.^11,23–25^ The inability of these studies to fully explain the sex differences in cognition and dementia risk may stem from unaccounted risk factors, geographical variations as well as differences in follow-up durations. Recent reports suggest that clusters of risk factors may better explain sex-related disparities in cognition and dementia.^26–28^

It remains unknown whether combinations of risk factors explain the sex differences in cognitive performance. Emerging evidence suggests that risk factors may differ between the sexes in prevalence and/or effect.^29,30^ A recent study on adults aged 20–76 years over a 12-year follow-up period supports this theory showing that different risk factors had a negative effect on memory performance among men and women, and these associations were influenced by age.^24^ Investigating to what extent dementia risk factors explain sex differences in cognitive performance and decline by adopting a multifactorial approach may improve our understanding of the differences in dementia risk between the sexes, and ultimately may unravel one of the most significant and unanswered questions in dementia research; ‘why is AD more prevalent among women?’.

This study aims to assess the association between sex and cognitive performance with repeated measures by (i) quantifying sex differences in multidomain cognitive scores, (ii) determining to what extent these risk factors contribute to the sex differences observed at baseline and in PEs, using repeat follow-up, and (iii) assessing the effect modification by age and genetic risk on sex differences in PEs. Consistent with previous reports we hypothesized that (i) sex differences will be observed in cognitive scores at baseline and over time, (ii) sex differences will only be partly explained by risk factors, (iii) sex differences will be greater in ‘older’ participants, and iv) sex differences will be more pronounced among Apolipoprotein ε4 (*APOE* ε4) carriers.

## Methods

### Study setting and population

The CHARIOT (Cognitive Health in Ageing Register: Investigational, Observational and Trial Studies in dementia Research) Prospective Readiness Cohort) Main Study (CPRO-MS) was a prospective, observational clinical study, conducted at Imperial College London.^31^ The study evaluated cognitive trajectories in cognitively healthy participants aged between 60–85 years. Participants were recruited through self-rereferral or from a community-based register. Of 987 participants screened, 964 participants had their baseline cognitive assessments completed. Individuals were deemed ineligible if they met any of the exclusion criteria (Supplemental Table 1).

If a participant's Repeatable Battery for the Assessment of Neuropsychological Status (RBANS) adjusted for age and education was 1.5 or more standard deviations (SD) below the population mean (normal), the participant was excluded from longitudinal follow-up unless adjudicated for inclusion by a medical monitor. Furthermore, to be eligible for the study, participants had to have a Clinical Dementia Rating of 0 (indicating cognitive normality) at baseline, which all participants fulfilled. Of 712 screenees eligible for follow-up and enrolled into the study, 691 were actively followed up post baseline.^31^ Scheduled assessments for data collection (Supplemental Table 2) occurred every six months (waves), and the median estimated participant time in the study was 18.1 months due to early study termination by the sponsor.

The study was carried out in accordance with Good Clinical Practice Guidelines (GCP), Guidelines for Good Pharmacovigilance Practices (GPP) issued by the International Society for Pharmacoepidemiology (ISPE), applicable national guidelines, and to the Declaration of Helsinki.^31^ The study has received National Research Ethics Serviced approval (15/L0/0711) and internal Imperial College London Research Ethics, Joint Research Compliance Office approval (JRCO: 15/1C/2791). All participants provided written informed consent before participating in the study. This study did not use human subjects and/or animals; we used anonymized data from the CPRO-MS.

### Outcome

The RBANS is a sensitive means of distinguishing healthy controls from very mild dementia.^32^ The test lasts approximately 20–30 min and is administered face-to-face. This composite battery consists of 12 subtests that measures five cognitive indices: immediate memory, visuospatial/constructional, language, attention, and delayed memory index. The sum of the five index scores was then used to calculate an age-adjusted total scale score. A lower RBANS total scale score reflects greater cognitive impairment. Change in RBANS total scale between baseline and at each six-monthly follow-up visit is the primary outcome for this study. Our secondary explorations are changes in RBANS cognitive indices between baseline and at each six-monthly follow-up visit. The internal consistency reliability of RBANS scale at baseline measured by Cronbach's α was 0.81.

### Genotype

Participants were directly genotyped to assess *APOE* status (ε4 allele carrier or non-carrier) using the Quest Pharmaceutical Services validated pyrosequencing genotyping assays for *APOE* codon 112T > C and codon 158C > T polymorphic variants. The direct measurement of genomic DNA samples ensures accurate genotyping.^31^ Measured *APOE* alleles did not deviate from the Hardy-Weinberg equilibrium (p > 0.05). Possessing the *APOE* ε4 allele increases susceptibility and accounts for up to 50% of the population attributable risk for sporadic late-onset AD.^33,34^

### Lifestyle measures

Data were collected from medical records by the investigator or self-reported by the participant. The participants were asked to complete the Imperial Lifestyle Questionnaire (ILQ) which consists of 106 items assessing and capturing a wide range of health and lifestyle characteristics. The ILQ was used to capture self-reporting data and used to determine demographics including age, sex (male/female), ethnicity (white/other) and marital status (single/married), socioeconomic status including education (number of years), occupation (manual work/intermediate/managerial & professional), annual personal and household income, smoking history status (current smoker/ex-smoker/non-smoker).

Daily consumption of alcohol (calculated as grams per day), fruit and vegetables (frequencies per day calculated as grams) was collected using the semi-quantitative Scottish Collaborative Group Food Frequency Questionnaire, SCQ-FFQ^35^ that has been validated among older adults in the UK. Derived from the dietary questionnaires used in the Scottish Heart Health/MONICA Study, the SCG-FFQ is a 150-item instruments designed to assess the habitual diet of UK residents over the past 3 months.^31^

Diagnosed health history was also assessed and included diabetes mellitus, cardiovascular disease (CVD), stroke, hypertension, and depression. Participant's height (cm) and weight (kg) were measured at clinic and these measurements were used to calculate body mass index (BMI, kg/m^2^) by dividing weight by height in meters squared. Physical activity was measured via a validated 12-item self-reported questionnaire; Physical Activity Measurement for the Elderly (PASE)^36^ designed to measure the amount of physical activity over the previous week in adults aged ≥65 years. Participants were asked to capture duration (<1 h/1–2 h/2–4 h/>4 h), frequency (never/seldom/sometimes/often) and intensity (low/moderate/vigorous) of leisure activities and exercises.

Individuals with missing values in one or more of the following variables were excluded: sex, age, and baseline RBANS indices (Supplemental Figure 1). Individuals with missing values in one or more of the following variables were excluded: sex, age, and baseline RBANS indices (Supplemental Figure 1). Little's test for missing completely at random (MCAR) was performed and the results indicated that the data was not MCAR (p = 0.07). After further investigation, we found that missingness in education was more likely among female respondents. Since participants were not instructed to leave questions blank in the ILQ, we consider the missing data pattern to be missing at random (MAR). We imputed data using multiple imputation to obtain 10 sets of coefficients and standard errors. Imputation was conducted on all the variables with missing data presented in the Supplemental Table 3.

### Statistical analysis

Participant characteristics by sex were described using chi-squared tests for categorical variables and independent sample *t*-tests for continuous variables. To compare cognitive age-adjusted scores between the sexes, both independent *t*-tests and Wilcoxon rank-sum tests were conducted. We assessed the association between cognitive performance (assessed via RBANS Index scores) at baseline and sex using multivariable linear regression analyses with progressive adjustment for covariates. We employed two approaches to assess the contribution of confounders in our analysis.

First, we grouped models (1, 2, and 3) to account for the combined effect of these confounders on the association between sex and cognitive performance. Model 1 (demographic model) was adjusted for ethnicity, Model 2 (socioeconomic model) was adjusted for years of education, annual personal income, marital status and occupation, Model 3 (vascular and lifestyle model) was adjusted for hypertension, diabetes, stroke, CVD, BMI, alcohol intake, consumption of fruit and vegetables, smoking status, physical activity score and depression. Second, we assessed the contribution of each factor individually to determine its specific contribution on the observed association in Supplemental Table 4.

Linear mixed models were used to assess the effect of sex on change in cognitive performance. We tested linear and non-linear trajectories by comparing linear and polynomial mixed-effects models. There results showed no significant improvement in fit with polynomial models, therefore a linear trajectory was used. We created a ‘time’ variable which is the difference between each visit date (wave) from the date of the baseline visit in years. We assessed the effect of sex and time interaction on change in cognitive trajectories adjusted for covariates. Random effects were specified for individual participants and time of follow-up at different waves from baseline. All analyses were done in Stata 18 and a two-sided p value of <0.01 was considered statistically significant given the analysis of multiple RBANS indices.

We examined the effect of sex and time interaction on change in cognitive trajectories in ‘younger’ (60–68 years) versus ‘older’ 69–85 years) participants based on the median age of participants in the CPRO-MS dataset. Additionally, we assessed the effect of sex and time on change in cognitive trajectories among ‘carriers’ of the *APOE* ε4 allele versus ‘non-carriers’ by firstly stratifying participants into two strata and then examining the two-way interaction of sex and time on change in cognitive performance.

Furthermore, the following sensitivity analyses were conducted: (i) we have completed all longitudinal analyses using data from baseline to wave 6 (excluding data from wave 7) to confirm that any inferences we make from our longitudinal analysis is not influenced by wave 7 given the significantly low number of participants who completed this visit (Supplemental Table 1^2^). (ii) We have completed all analyses using complete case data to ensure our findings are robust to the imputation method used (Supplemental Tables 6 and 9).

**Table 2.** Multiple regression analyses with progressive adjustment for confounders to assess the association between sex and cognitive performance in the RBANS indices at baseline, N = 964.

Model No.	*Difference in mean scores * men compared to women (ref.) (95% CI)	p	*Difference in mean scores * men compared to women (ref.) (95% CI)	p
	Total Scale		Visuospatial/constructional	
1	−3.5 (−5.2, −1.9)	<0.001	4.5 (2.5, 6.4)	<0.001
2	−5.3 (−7.0, −3.6)	<0.001	3.9 (1.8, 6.0)	<0.001
3	−5.4 (−7.2, −3.6)	<0.001	4.2 (2.0, 6.5)	<0.001
	Immediate Memory		Delayed Memory	
1	−5.6 (−7.4, −3.8)	<0.001	−3.3 (−4.8, −1.8)	<0.001
2	−7.3 (−9.2, −5.4)	<0.001	−4.4 (−5.9, −2.8)	<0.001
3	−7.7 (−9.7, −5.8)	<0.001	−4.7 (−6.3, −3.0)	<0.001
	Language		Attention	
1	−6.9 (−8.5, −5.4)	<0.001	−0.8 (−2.8, 1.2)	0.5
2	−8.0 (−9.7, −6.4)	<0.001	−2.7 (−4.9, −0.6)	0.01
3	−8.4 (−10.2, −6.6)	<0.001	−2.6 (−4.9, −0.4)	0.02

Model 1: ethnicity; Model 2: as for model 1 plus years of education, personal annual income, occupation & marital status; and Model 3: as for model 2 plus CVD, hypertension, diabetes, stroke, BMI, smoking status, alcohol intake, food & vegetable consumption, physical activity & depression. Significance level was set at 0.01.

## Results

### Study population

Of 964 participants assessed with RBANS at baseline and 691 participants at follow-up, the mean (SD) age was 68.8 (3.9) years. Participant characteristics are presented by sex in Table 1. Among all baseline participants, 528 (54.8%) participants were female, and nearly all participants were white (87.2%). Compared with women, men reported higher annual household and personal incomes (p < 0.001),held higher managerial professions (p < 0.001), and spent more years in education (p = 0.02). Men were also more likely to have hypertension (p < 0.001), diabetes (p = 0.002) and CVD (p < 0.001). Men had higher BMI (p = 0.004) and were more likely to be smokers (p < 0.001) and consume more alcohol daily (p < 0.001). Women were more likely to consume more fruit (p = 0.02) and vegetables (p = 0.03). Men had significantly lower scores than females in baseline global cognition (p < 0.001), as well as tests assessing immediate memory (p < 0.001), language (p < 0.001) and delayed memory (p < 0.001). We additionally conducted the Wilcoxon rank sum test to assess cognitive scores between the sexes and results were similar to our independent samples t-test (Supplemental Table 4). Cohen's d for the RBANS total scale was 0.3, indicating a small to medium mean difference between men and women. Supplemental Table 6, summarizes the Cohen's U3 values for the RBANS indices, indicating the proportion of females who scored above the age-adjusted mean of males at baseline. Cohen's U3 values ranged from 0.3 to 0.6 across different domains, with the higher proportion (60%) of females scoring above men in the visuospatial/constructional index.

**Table 1.** Population characteristics by sex at baseline, N = 964 and follow-up, N = 691.

	Baseline cohort					Follow-up cohort			
Cohort	All	Women	Men	P	Cohen's d	All	Women	Men	p
N	964	528 (54.8%)	436 (45.2%)			691	396 (57.4%)	294 (42.6%)	
*Demographics*									
Age (y), Mean (SD)	68.8 (3.9)	68.7 (3.9)	68.8 (3.9)	0.7		68.8 (3.8)	68.8 (3.8)	68.7 (3.8)	0.8
Ethnicity, n (%)				0.8					0.9
White	841 (87.2%)	465 (88.1%)	376 (86.2%)			627 (90.9%)	363 (91.9%)	264 (89.2)	
Other	74 (7.7%)	40 (10.2%)	34 (7.8%)			34 (4.9%)	20 (5.1%)	14 (4.7%)	
*Socioeconomic*									
Education (y), Mean (SD)	15.1 (3.01)	14.9 (2.9)	15.4 (3.1)	0.02		15.1 (3.1)	15.0 (2.9)	15.3 (3.1)	0.1
Income (£), Mean (SD)									
Annual personal	35,242 (17,271.5)	30,148.5 (14,274.9)	40,622.6 (18,504.5)	<0.001		35,308.9 (17,137.3)	29,842.1 (13,728.2)	41,706.5 (18,487.3)	<0.001
Annual household	49,878.2 (28,434.2)	43,149.4 (24,463.9)	56,894.5 (30,543)	<0.001		50,634.8 (28,061.2)	44,070.1 (24,518.4)	58,213.2 (29,965)	<0.001
Occupation, n (%)				<0.001					<0.001
Manual Work	67 (7%)	22 (4.2%)	45 (10.3%)			44 (6.4%)	14 (3.5%)	30 (10.1%)	
Intermediate	246 (26%)	190 (36%)	56 (12.8%)			183 (26.5%)	149 (37.7%)	34 (11.5%)	
Managerial & professional	545 (57%)	246 (46.6%)	299 (68.6%)			387 (56.1)	180 (45.6%)	207 (69.9%)	
Marital status, n (%)				<0.001					<0.001
Single	404 (42%)	269 (66.6%)	135 (33.4%)			278 (40.3%)	193 (48.9%)	85 (28.7%)	
Married	555 (58%)	258 (46.5%)	297 (53.5%)			409 (59.3%)	201 (50.9%)	208 (70.3%)	
*Cardiovascular*									
Hypertension, n (%)	329 (34.1%)	145 (44.1%)	184 (55.9%)	<0.001		222 (32.2%)	101 (25.6%)	121 (40.9%)	<0.001
Diabetes, n (%)	72 (7.5%)	27 (37.5%)	45 (62.5%)	0.002		47 (6.8%)	21 (5.3%)	26 (8.8%)	0.07
Stroke, n (%)	11 (1.1%)	7 (63.6%)	4 (36.4%)	0.6		9 (1.3%)	5 (1.3%)	4 (1.4%)	0.9
CVD, n (%)	187 (19.4%)	80 (42.8%)	107 (57.2%)	<0.001		146 (21.2%)	65 (16.5%)	81 (27.4%)	0.001
*Lifestyle*									
BMI (kg/m2), Mean (SD)	26.0 (4.4)	25.6 (4.8)	26.5 (3.7)	0.004		25.9 (4.3)	25.5 (4.8)	26.3 (3.5)	0.02
Alcohol (g), Mean (SD)	13.2 (14.7)	9.8 (11.4)	17.2 (17.1)	<0.001		13.9 (15.1)	10.5 (11.8)	18.3 (17.6)	<0.001
Fruit (g), Mean (SD)	270.5 (247.3)	289 (197.3)	249.1 (293.7)	0.02		263 (231.3)	279.6 (187.5)	242.4 (275.1)	0.05
Vegetables (g), Mean (SD)	165.3 (154.6)	176.6 (138.9)	152.4 (169.9)	0.03		163.4 (159.4)	170.4 (126)	154.8 (192.4)	0.2
Smoking status, n (%)				<0.001					<0.001
Non-smoker	506 (52.5%)	307 (60.7%)	199 (39.3%)			362 (52.5%)	232 (58.7%)	130 (43.9%)	
Ex-smoker	385 (39.9%)	183 (47.5%)	202 (52.5%)			279 (40.4%)	135 (34.2%)	144 (48.6%)	
Smoker	63 (6.5%)	30 (47.5%)	33 (52.4%)			42 (6.1%)	22 (5.6%)	20 (6.8%)	
Physical Activity (hours), Mean (SD)	7.4 (1.8)	7.4 (1.8)	7.4 (1.9)	1		7.5 (1.8)	7.5 (1.7)	7.5 (1.8)	0.9
Depression, n (%)	81 (8.4%)	51 (63%)	30 (37%)	0.1		65 (9.4%)	42 (10.6%)	23 (7.8%)	0.2
*Cognitive performance*									
RBANS score, Mean (SD)									
Total Scale	102.8 (13.8)	104.4 (13.8)	100.8 (13.4)	<0.001	0.3	106.4 (12.1)	107.2 (12.7)	105.2 (11.3)	0.03
Visuospatial/constructional	98.4 (15.9)	96.4 (16.1)	110.8 (15.4)	<0.001	−0.9	100.4 (14.3)	98.2 (14.3)	103.4 (13.8)	<0.001
Immediate Memory	103 (14.8)	105.6 (13.6)	99.9 (15.5)	<0.001	0.4	106.4 (12.6)	107.7 (12.5)	104.6 (12.5)	0.001
Delayed Memory	100.5 (12.0)	102.1 (11.5)	98.7 (12.4)	<0.001	0.3	103.3 (9.6)	104 (9.7)	102.4 (9.3)	0.03
Language	104.3 (13.3)	107.5 (13.7)	100.5 (11.7)	<0.001	0.6	106.4 (11.9)	109.1 (12.5)	102.8 (9.8)	<0.001
Attention	104.1 (15.9)	104.5 (16.2)	103.7 (15.5)	0.4	0.05	106.9 (15.1)	10 7 (15.3)	106.7 (14.9)	0.8
APOE ε4 Carrier, n (%)						164 (23.8%)	93 (23.5%)	71 (24%)	0.9
ε2/ε4, n (%)						22 (3.2%)	13 (3.3%)	9 (3.1%)	–
ε3/ε4, n (%)						137 (19.9%)	78 (19.7%)	59 (20.1%)	–
ε4/ε4, n (%)						5 (0.7%)	2 (0.5%)	3 (1%)	–

Independent t-tests were used for continuous variables and chi-square tests for categorical variables. Categorical variables are present as N (%) and continuous variables as mean (standard deviation).

Sex differences in demographic, lifestyle and vascular conditions were similar to our findings described above when comparing characteristics by sex in those included at baseline and in the follow-up cohort (>18 months follow-up). 164 (23.8%) participants were carriers of the *APOE* ε4 allele in the longitudinal cohort, but no sex differences were observed in *APOE* ε4 carrier status. Sex differences between the baseline and follow-up cohorts in cognitive performance were also similar but mean scores were greater in the follow-up cohort as participants who scored 1.5 or more SD below the population mean (normal), were excluded from longitudinal follow-up (Supplemental Figure 1).

### Sex differences in cognitive performance at baseline

Table 2 shows the results of cross-sectional analysis assessing the association between sex and cognitive performance in the RBANS adjusting for covariates using three models: Model 1 adjusts for demographic variables; Model 2 further adjusts for socioeconomic factors; and Model 3 further adjusts for vascular and lifestyle risk factors. Sex differences at baseline (Table 1) is the base model (Model 1) additionally adjusted for ethnicity, favored women in the RBANS total scale (mean difference = −3.5, 95%CI: −5.2, −1.9), immediate memory (mean difference = −5.6, 95%CI: −7.4, −3.8), delayed memory (mean difference = −3.3, 95%CI: −4.8, −1.8) and language index (mean difference = −6.9, 95%CI: −8.5, −5.4) and favored men in the visuospatial/constructional index (mean difference = 4.5, 95%CI: 2.5, 6.4). There was no significant association between sex and cognitive performance in the attention index.

Upon additional adjustment for socioeconomic factors and marital status (Model 2) found men scored worse in the RBANS total scale (mean difference: −5.3, 95%CI: −7.0, −3.6), immediate memory (mean difference: −7.3, 95%CI: −9.2, −5.4), delayed memory (mean difference: −4.4, 95%CI: −5.9, −2.8), language index (mean difference: −8.0, 95%CI: −9.7, −6.4) and attention index (mean difference: −2.7, 95%CI: −4.9, −0.6) compared to women. The magnification of the association conveys an underestimation of the sex difference in these indices due to the (negative) confounding effects of education, income, occupation, and marital status. However, the sex difference in visuospatial/constructional index attenuated suggesting that socioeconomic factors may explain some of these differences (mean difference: 3.9, 95%CI: 1.8, 6.0).

Additional adjustment for vascular and lifestyle risk factors in Model 3 further intensified the sex difference unfavorable to men in the RBANS total scale (mean difference = −5.4, 95%CI: −7.2, −3.6), immediate memory (mean difference = −7.7, 95%CI: −9.7, −5.8), delayed memory (mean difference = −4.7, 95%CI: −6.3, −3.0), and language index (mean difference = −8.4, 95%CI: −10.2, −6.6). The additional adjustment of vascular and lifestyle risk factors in Model 3, had negligible effects on estimates in the visuospatial/constructional and attention index implying the small contribution of these factors on sex difference in these RBANS subtests. This small change in estimates further reinforces the robustness of sex differences observed in these cognitive domains.

We assessed change in magnitude of the association between sex and cognitive performance at baseline with the inclusion of single AD dementia risk factors (Supplemental Table 5). Annual household income was a significant confounder in our sample as the sex difference (unfavorable to men) was magnified in the total scale and all RBANS indices upon its inclusion. Risk factors like personal annual income, consumption of alcohol, fruit and vegetables were also considered significant confounders in our sample but in domain specific indices.

Results in Supplemental Table 7 shows the results from all-case models assessing the association between sex and cognitive performance in the RBANS at baseline.

### Follow-up cohort

In longitudinal analyses, PEs (increase in cognitive scores per year) were observed in the RBANS total scale and all indices except for the visuospatial/constructional index where we observed a decline in total scores, and the language index which showed no significant change per year (Supplemental Table 8).

**Figure 1.**
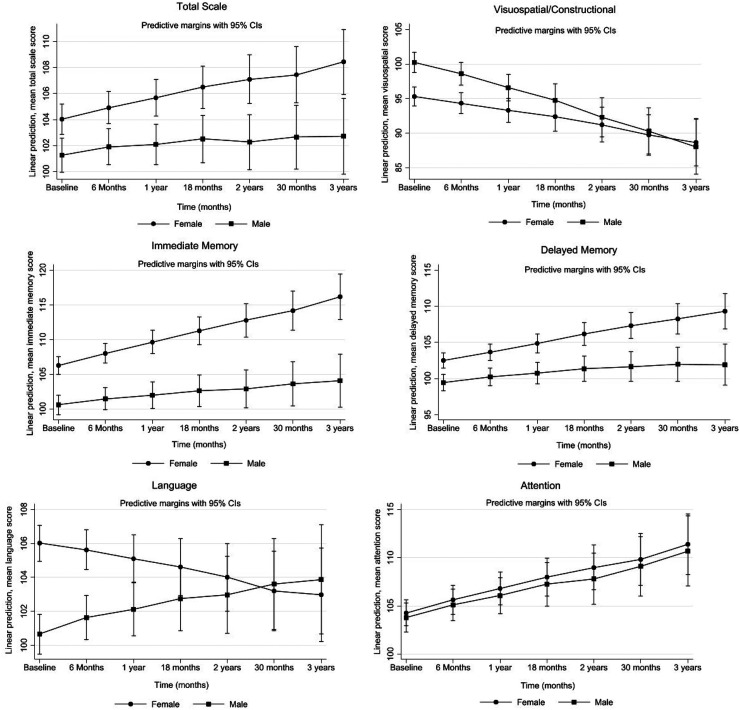
Projected mean changes in RBANS scores: total scale, visuospatial/constructional, immediate memory, delayed memory, language and attention index with 95% confidence error bars over time by sex. Linear mixed models were used to assess change in participants’ cognitive performance over time, by modelling the interaction between sex and time on change in cognitive trajectories. Model was adjusted for ethnicity, years of education, annual personal income, occupation, marital status, CVD, hypertension, diabetes, stroke, BMI, smoking status, daily alcohol consumption, physical activity, and depression, N = 691.

The effect of time and sex on cognitive performance in the RBANS, fully adjusted for AD dementia covariates are shown in Figure 1 and Table 3. Over time, we observed a significant sex difference in PEs in the RBANS total scale with men showing less PEs (Table 3). The change in mean scores per year for men was 1.3 points lower in the total scale (95%CI: −2.1, −0.5). Men also showed less PEs in both tests of memory and in the visuospatial/constructional index adjusted for AD dementia covariates. The change in mean scores per year for men was lower by 1.8 points in immediate memory (95%CI: −2.9, −0.8), 1.2 points lower in the delayed memory (95%CI: −2.0, −0.4) and 1.6 points lower in the visuospatial/constructional index (95%CI: −2.8, −0.4) compared to women. In contrast, sex differences in the language index favored men as they scored 1.1 points higher (95%CI: 0.02, 2.1) over time compared to women although this was not statistically significant. We did not observe sex differences in PEs in the attention index.

**Table 3.** Linear mixed model analyses to assess the interaction between sex and time on the mean change in cognitive trajectories of the RBANS per year, N = 691.

Ref: men compared to women	β (95% CI)	p	β (95% CI)	p
Total Scale			Visuospatial/Constructional	
Model 3: Main effect: Sex^a^	−2.6 (−4.4, −0.8)	0.005	5.2 (3.1, 7.4)	<0.001
Interaction between sex & time^b^	−1.3 (−2.1, −0.5)	0.002	−1.6 (−2.8, −0.4)	0.009
Immediate Memory			Delayed Memory	
Model 3: Main effect: Sex^a^	−4.5 (−6.3, −2.7)	<0.001	−1.6 (−3.0, −0.08)	0.04
Interaction between sex & time^b^	−1.8 (−2.9, −0.8)	0.001	−1.2 (−2.0, −0.4)	0.002
Language			Attention	
Model 3: Main effect: Sex^a^	−5.5 (−7.2, −3.9)	<0.001	−2.0 (−4.4, 0.3)	0.09
Interaction between sex & time^b^	1.1 (0.02, 2.1)	0.05	−0.6 (−1.5, 0.4)	0.2

Adjusted for ethnicity, years of education, personal annual income, occupation, marital status, CVD, hypertension, diabetes, stroke, BMI, smoking status, alcohol consumption, physical activity & depression.

^a^ Difference in mean scores, men compared to women.

^b^ Change in mean scores per year in men compared to women (practice effects). r: correlation between the random intercept and linear slope. Significance level was set at 0.01.

These differences between the sexes were unexplained by the AD dementia covariates we adjusted for in our sample: Results from models 1 and 2 (provided in Supplemental Table 9) were similar to the fully adjusted model 3 results displayed below in Table 3.

In the RBANS total scale and attention index we noted a positive moderate correlation (r = 0.3), this indicates that individuals with higher baseline scores. Similarly, this correlation was noted in both tests of memory (immediate memory r = 0.07 & delayed memory r = 0.1), although this relationship is not very strong. In the visuospatial/constructional index we observed a moderate negative correlation (r = −0.4). This suggests that individuals with higher baseline scores in this domain tend to experience faster decline overtime, whereas those with lower scores had slower decline or may even improve slightly overtime. We also found a perfect correlation (r = 1) in the language index, indicating that performance at baseline, predicted the rate of change. Results were similar in the complete cases analysis (Supplemental Table 10) and exclusion of wave 7 cognitive data from our analysis did not affect our results (Supplemental Table 1^1^)

### Stratified analysis for non-modifiable risk factors

**Figure 2.**
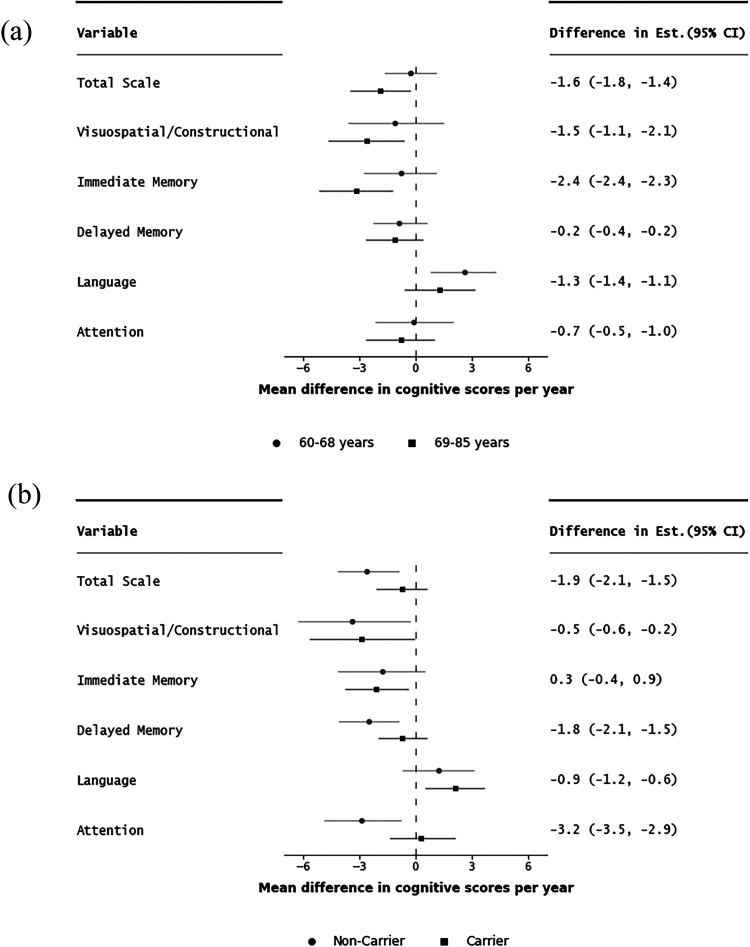
Mean difference in change in RBANS scores between men and women per year (interaction between sex and time) stratified by age category (a); ‘younger’ (60–68 years) versus ‘older’ (69–85 years) and *APOE* ε4 carrier status (b); ‘carrier’ versus ‘non-carrier’ of the ε4 allele. Difference (older-younger) and (Carrier-Non-carrier) in mean estimate and error bars showing difference in 95% confidence intervals. Reference: men compared to women.

We investigated whether PEs in mean RBANS scores differed between the sexes over time (per year) while stratifying for age (younger (60–68 years) versus older (69–85 years) strata) and then *APOE* ε4 carrier status (carrier versus non-carrier) in Figure 2 and Supplemental Tables 12 and 13.

Greater sex differences over time were observed among the older group favoring women in the RBANS total scale (mean difference in change per year: −1.9; 95%CI: −3.5, −0.3 for older versus −0.3; 95%CI: −1.7, 1.1 for younger) and the following subtests: visuospatial/constructional (−2.6; 95%CI: −4.7, −0.6 for older versus −1.1; 95%CI: −3.6, 1.5 for younger) and immediate memory index (−3.2; 95%CI: −5.2, −1.2 for older versus −0.8; 95%CI: −2.8, 1.1) for younger). By contrast, greater sex differences were observed among the ‘younger’ age strata favoring men in the language index (mean difference: 2.6; 95%CI: 0.8, 4.3 for younger versus 1.3; 95%CI: −0.6, 3.2 for older).

Sex differences over time were greater among carriers of the *APOE* ε4 allele favoring women in the RBANS total scale (mean difference in change per year : −2.6; 95%CI: −4.2, −0.9 for carriers versus −0.7; 95%CI: −2.1, 0.7 for non-carriers), and in the following subtests: delayed memory (−2.5; 95%CI: −4.1, −0.9 for carriers versus −0.7; 95%CI: −2.0, 0.6 for non-carriers) and attention index (mean difference in change per year: −2.9; 95%CI: −4.9, −0.8 for carriers versus 0.3; 95%CI: −1.4, 2.1 for non-carriers) (Figure 2).

We additionally assessed sex differences stratified by *APOE* ε4 carrier status excluding carriers of the ε2 allele, given that the possession of this allele confers some protection against cognitive decline and dementia risk (Supplemental Table 14). Similar, to our findings above, we found that sex differences were greater among carriers of the ε4 allele and men performed worse than women.

## Discussion

In a large London population-based sample we assessed sex differences in cognitive scores cross-sectionally, finding that women had better scores in almost all RBANS indices except for the visuospatial index which men outperformed women. In the attention index, we did not observe any differences. The contribution of differences in lifestyle and health risk factors to observed sex differences was negligible, however differences were magnified after taking into account greater levels of income, and education among men as well as occupation and marital status. The female advantage persisted in greater PEs (i.e., improvement in cognitive scores) in the total scale, visuospatial/constructional, immediate and delayed memory index in longitudinal analyses of cognitively healthy participants with up to three years of multidomain cognitive follow-up.

Similar to our findings, studies on cognitively healthy adults have also reported that at baseline men performed better in the visuospatial index while women outperformed men in most cognitive domains including memory.^11,37^ The female advantage observed can also be explained by their ability to access and date their memories more efficiently^38,39^ compared to men. In our sample, risk factors (such as socioeconomic factors), were classic examples of negative confounders as they potentially masked and/or underestimated the true sex differences in cognitive trajectories, thus highlighting their pivotal role on the association between sex and cognitive performance.

The RBANS scores among the CPRO-MS participants fell within the range considered cognitively healthy. PEs were detected in most RBANS indices, including the total scale. PEs were more pronounced in women after repeated tests, which was evident in the RBANS total scale, and both memory tests. These findings are consistent with a study by Zheng et al. (2020)^20^ using the CPRO-SubStudy (SS), a separate biomarker enriched prospective longitudinal study now in its 5 year follow-up period. Participants also scored better in the attention index over time, with the sex differences being more subtle. In the language index, the sex difference and PEs were more pronounced among men, similar to findings from a study of cognitively normal adults.^40^ The lower PEs observed mostly in men may indicate potential cognitive decline, as previous studies have linked reduced PEs across repeated cognitive tests to subsequent cognitive decline and an increased risk of MCI and/or dementia.^20,41,42^ This implies that men may be more likely to be diagnosed with dementia sooner than women who exhibited higher levels of PEs.

Our observation of greater PEs in women following repeat assessments over 3 years, can be considered in line with previous studies conducted using UK based datasets^37,43^ that similarly, observed slower rates of decline over time among women compared to men including the immediate memory and the visuospatial/constructional index. A study using data from two large prospective cohort studies in the UK reported slower rates of decline in memory among women after adjustment for educational attainment.^37^ The sex differences in cognitive scores per year remained after adjusting for covariates which is in line with a large study of adults aged ≥50 years in the UK^43^ and a study that used data from two large Dutch ageing cohorts, both of which also noted sex differences favoring women after adjustment for education level.^44^ However, previous studies investigating sex differences in cognition have been inconsistent with some reporting steeper levels of decline among women^23,25,44^ and some reporting no differences over time.^45–48^ Several reasons may explain why our results differ from previous studies, including ethnic, geographical and cultural diversity of different cohorts that influence the lifetime exposure of an individual, in addition to the effect of sex. As an example, in a study conducted in Japan, the steeper levels of decline in women were explained by differences in levels of education and occupation.^25^ Additionally, differences in measures of time, duration of repeated measures and the outcome will likely have implications on results. For example, while our data had a median follow-up of 1.5 years in a study with over 23 years of follow-up, women showed up to 10% faster decline in cognitive domains compared to men.^44^ Similarly, in a study with a median follow-up of 7.9 years,^23^ faster rates of decline among women were found in global cognition and executive function.

In our study, we adjusted for a wide range of established AD dementia risk factors. However, the contribution of these risk factors to differences in multidomain cognitive scores was small. We cannot rule out, that other unassessed risk factors such as hearing loss, air pollution, traumatic brain injury and social isolation may contribute to some of the observed differences.^21,22^ Furthermore, while well-established risk factors for dementia have been identified, their impact on ‘normal’ cognitive decline is not yet fully understood. Distinguishing between normal cognitive decline due to ageing and dementia can aid the development of targeted interventions as well as our understanding of cognitive function across the lifespan.^49^ Finally, differences in multidomain cognitive performance may be driven by an inherent biological mechanism and therefore may not be explained by the risk factors explored. Along these lines, it is proposed^50,51^ that women may possess a form of cognitive reserve in relation to performance in memory which is key in the clinical diagnosis of MCI and dementia regardless of AD clinical presentation.^52,53^ If women indeed possess greater levels of cognitive reserve compared to men, it is plausible that current cognitive tests may not be sensitive enough to accurately detect and capture subtle cognitive changes between the sexes. Another factor to consider is that current risk factors for dementia may have a differential impact on cognitive performance in men compared to women.

Age-stratified analysis showed greater sex differences among the ‘older’ age group in two out of the five cognitive domains measured including the immediate memory, and visuospatial/constructional index, withmen scoring worse compared to women. Our results support previous studies that reported higher prevalence of AD among participants aged ≥80 years of age^4,54–56^ and a study that observed larger sex differences in memory among participants aged ≥ 70 years.^37^ A systematic review noted that differences in cognitive performance remained stable in individuals aged between 60–80 years, but in participants aged >80 years sex differences in cognitive decline became apparent.^57^ It is possible that although we observed sex differences in age-stratified analysis, given our relatively young sample in the CPRO-MS, the observed sex differences in cognitive scores may have been underestimated for the oldest old.

Sex differences were more pronounced among carriers of the *APOE* ε4 allele in three out of the five cognitive domains measured, with men scoring worse in the RBANS total scale, delayed memory and attention index. Similarly, a study using an extremely large UK based cohort, reported a significant association between *APOE* ε4 carriage and cross-sectional cognitive scores although the effect size was small and the interactions with sex and *APOE* genotype was suggestive with male carriers scoring worse.^58^ Whilst in our sample, we observed greater sex differences favorable to women, studies by Fleisher et al. (2005) and Mortensen et al. (2001) who investigated the relationship between *APOE* genotype (carrier versus non-carrier) and sex (men versus women) recorded greater decline in memory between *APOE* ε4 carriage and women compared to men.^59,60^ This discrepancy may be explained, by the older age group, relatively small sample size (N > 200) and the inclusion of subjects with MCI. Fleisher and colleagues conducted their analysis on subjects with MCI,^59^ whereas our sample included cognitively healthy subjects. Mortensen and colleagues also used cognitive healthy individuals but observed greater decline in women who were carriers of the and aged 70–80 years.

### Strengths and limitations

Major strengths of our study are a large sample size, repeated measures of a validated and sensitive cognitive assessment as well as a robust adjustment for a wide range of well-established AD dementia risk factors including lifestyle, socioeconomic and vascular factors that were assessed using standardized measures and validated tools. Furthermore, the study recruited community-based residents, aiming to achieve a sample reasonably representative of the wider London population.^61^

However, there are several potential limitations to note. First, our sample mainly consisted of participants who were of white ethnicity. This limits the generalizability of our findings to a wider range of ethnicities. Additionally, our findings are limited to a specific geographical region. Location is a key variable to consider as it can influence cultural and environmental exposures and is also one of the explanations for discrepancies in dementia risk.^62^ Second, individuals who volunteer to participate in research are often more likely to be white, healthier, better educated and socioeconomically well-off creating volunteer bias.^63^

The strongest predictor of dementia and cognitive impairment is advancing age. The CPRO-MS consisted of a relatively ‘younger’ cohort as our sample consisted of only 18 participants who were aged >80 years. Additionally, we were not able to investigate the birth cohort effect on differences in cognitive scores between the sexes. Considering and incorporating birth cohort in the study of sex differences is essential as gradients of social inequalities between the sexes in the UK has evolved through time. For example, women have more access to higher education and higher occupational positions to develop intellectual experiences through occupation.

Furthermore, participants were cognitively healthy at baseline and the follow-up time in this study was extremely short. Long-follow-up periods for dementia studies is essential to observe changes in cognitive performance and disease progression. A follow-up period of up to 3 years was not sufficient to observe significant changes in performance and to counteract the influence of PEs. Likewise, we were not able to assess the intermediate phase of MCI as the study only included cognitively healthy participants and with a relatively short follow-up. Future studies should account for the intermediate phase of MCI as a distinct category to assess potential sex differences in all stages of cognitive impairment. Lastly, while in our study we attempted to adjust for a plethora of covariates we cannot exclude the effect of residual confounding and the impact of some measured and unmeasured covariates including hormone use and other factors such as sleep and anxiety.

### Conclusion

These results demonstrate significant sex differences favoring women in cognitive functions at baseline and in PEs (trajectories over time). Well-established AD dementia covariates had minimal contribution to the sex differences we observed. Future studies should assess effect modification by sex when investigating sex differences in cognitive function and dementia, incorporating sex-specific risk factors such as reproductive history, number of pregnancies and the use of hormone replacement therapy to capture the full spectrum of exposures related to women. Unravelling sex differences in cognitive outcomes as well as their contributing risk factors may contribute to understanding differences in cognition and AD dementia prevalence between the sexes. Additionally, future studies investigating effect modification of non-modifiable risk factors by sex including *APOE* genotype should consider assessing various combinations to accurately determine its effect on cognition, given that different variations of the allele confer varying levels of risk on developing AD.

Women consistently outperforming men in cognitive tests at baseline and over time suggests that they may be under-diagnosed for AD, as the clinical onset of AD may be more insidious, leading to delayed diagnosis and treatment. Diagnosing dementia in women is complicated due to their atypical symptoms and slower onset of cognitive decline. Tailoring assessment tools by sex may aid the development of tests that are designed to detect subtle changes in cognitive function and performance that may indicate underlying brain changes or cognitive decline in both sexes. This is particularly important as women may exhibit different patterns of decline compared to men.

Longitudinal assessments should consider the influence of PEs on cognitive decline patterns. Cognitive assessments should incorporate gender and/or sex-specific diagnostic criteria that considers potential differences in symptom presentation, PEs and disease progression in women. Developing assessments that are more sensitive to these differences may facilitate earlier detection of cognitive decline.

Despite the recent focus on modifiable risk factors for dementia, the role of sex as a significant predictor of AD has been largely overlooked in dementia research. A clearer understanding of the interactions between sex and the gender variables with age throughout the life course can potentially yield strategies that can prevent or target risk factors specific to each sex.

## Supplemental Material

sj-docx-1-alz-10.1177_13872877251339833 - Supplemental material for Sex differences in cognitive trajectories and practice effects in a cohort of older Londoners: The role of risk factors Supplemental material, sj-docx-1-alz-10.1177_13872877251339833 for Sex differences in cognitive trajectories and practice effects in a cohort of older Londoners: The role of risk factors by Sima Toopchiani, Shireen Sindi, Neil Poulter, Sujin Kang, Chi Udeh-Momoh, Geraint Price, Miia Kivipelto, Lefkos Middleton and Oliver Robinson in Journal of Alzheimer's Disease
