# Supplementary material for: Sex differences in cognitive trajectories and practice effects in a cohort of older Londoners: The role of risk factors
Source: J Alzheimers Dis. 2025 May 7;106(1):230–44. doi: 10.1177/13872877251339833 (PMC12231851; doi:10.1177/13872877251339833)
Supplement: sj-docx-1-alz-10.1177_13872877251339833 - Supplemental material for Sex differences in cognitive trajectories and practice effects in a cohort of older Londoners: The role of risk factors [file sj-docx-1-alz-10.1177_13872877251339833.docx]

**Supplemental Material**

**Sex differences in cognitive trajectories and practice effects in a cohort of older Londoners: The role of risk factors**

**Supplemental Table 1.** Exclusion criteria for the CPRO-MS.^1^

| - Previous diagnosis of dementia, MCI or other degenerative neurological conditions. |
| --- |
| - Met criteria of AD at baseline (per National Institute on Ageing Alzheimer’s Association). |
| - History of traumatic brain injury, stroke, or transient ischaemic attack (TIA). |
| - Epileptic seizures: significant psychiatric illness. |
| - History of hydrocephalus. |
| - Uncontrolled hypothyroidism or hyperthyroidism. |
| - History of clinically significant unstable illness, metabolic conditions, or nutritional deficiencies. |
| - Clinically significant infections in the past 30 days, HIV positive, history of alcohol or drug dependence or abuse; memantine or cholinesterase medications such as sedatives, anticonvulsants, or pain medications; history of significant sensory or motor dysfunction, physical disability, that would hinder study participation; concurrent participation in another trial. Participants with age- and education-adjusted cognitive performance more than 1.5 standard deviation (SD) below normal or any indices of the RBANS were excluded as considered to have MCI. |

**References**

1. Udeh-Momoh C, Price G, Ropacki MT, et al. prospective evaluation of cognitive health and related factors in elderly at risk for developing Alzheimer's dementia: A longitudinal cohort study. *J Prev Alzheimers Dis* 2019; 6: 256-266.

**Supplemental Table 2.** CPRO-MS Baseline/screening visit data collection variables.

| **Demographics** | **Medical history** | **Neuropsychological testing** | **Self-reporting questionnaires** |
| --- | --- | --- | --- |
| Date of birth/Age | Cerebrovascular accident | Repeatable Battery for the Assessment of Neuropsychological Status | Depression |
| Sex | Confirmed myocardial infarction and symptoms of angina |  | Nutrition |
| Ethnicity | Thrombotic conditions |  | Physical activity |
| Marital status | Confirmed myocardial infarction and symptoms of angina |  | Health status |
| Education level | Congestive heart failure |  | Current and past smoking and alcohol consumption |
| Occupation | Hypertension |  |  |
| Occupation history | Diabetes |  |  |
| Personal annual income |  |  |  |
| Household annual income |  |  |  |

**Supplemental Figure 1.** Flow chart showing the number of participants included in the baseline and follow-up analysis.


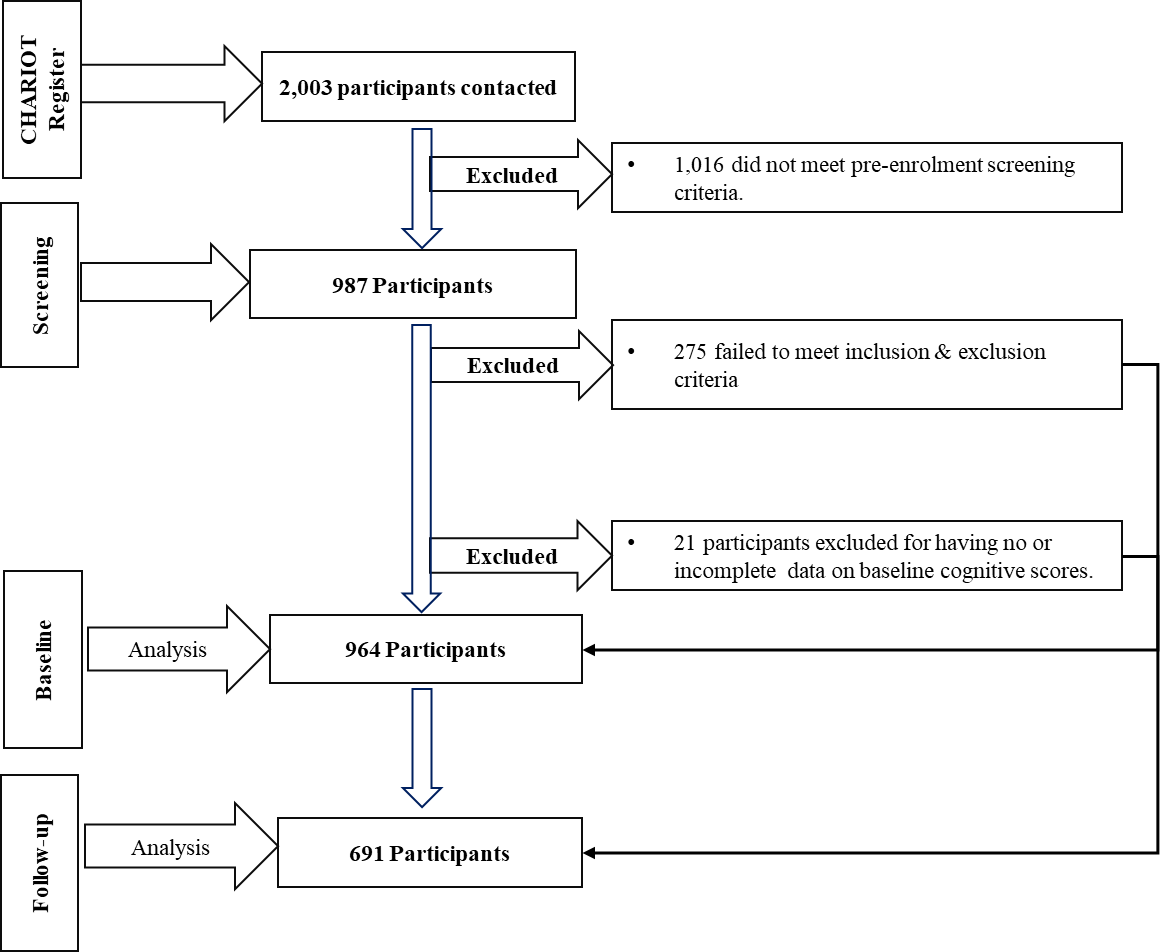


**Supplemental Table 3.** Number and % of missing variables at baseline for particiapnts.

| **Variable** | **N** | **n=missing** | **% missing** |
| --- | --- | --- | --- |
| ***Age at baseline*** | 964 | 0 | 0 |
| ***Ethnicity*** | 915 | 49 | 5.1 |
| ***APOE ε4 (carrier status)*** | 691 | 273 | 29.8 |
| ***Years of education*** | 743 | 221 | 32.0 |
| ***Annual Personal (£)*** | 839 | 125 | 16.8 |
| ***Household (£)*** | 813 | 151 | 18.0 |
| ***Occupation*** | 858 | 106 | 13.0 |
| ***Marital status*** | 959 | 5 | 0.6 |
| ***BMI (kg/m2)*** | 80 | 84 | 8.8 |
| ***Hypertension*** | 964 | 0 | 0.0 |
| ***Diabetes*** | 964 | 0 | 0.0 |
| ***Stroke*** | 964 | 0 | 0.0 |
| ***CVD*** | 964 | 0 | 0.0 |
| ***Smoking status*** | 954 | 10 | 1.0 |
| ***Physical Activity (h)*** | 867 | 97 | 10.2 |
| ***Alcohol (grams, g)*** | 858 | 106 | 12.2 |
| ***Fruit (grams, g)*** | 827 | 137 | 16.0 |
| ***Vegetables (grams, g)*** | 816 | 148 | 17.9 |
| ***Depression*** | 964 | 0 | 0.0 |
| ***Immediate Memory*** | 964 | 0 | 0.0 |
| ***Visuospatial*** | 964 | 0 | 0.0 |
| ***Language*** | 964 | 0 | 0.0 |
| ***Attention*** | 964 | 0 | 0.0 |
| ***Delayed Memory*** | 964 | 0 | 0.0 |
| ***Total Scale*** | 964 | 0 | 0.0 |

**Supplemental Table 4.** Presents the results of Wilcoxon rank-sum tests examining differences in cognitive performance between the sexes.

| **Total Scale** |  | **N** | **Rank Sum** | **Expected** | **Z-statistic** | **Exact p (Wilcoxon)** | **p (t-test)** |
| --- | --- | --- | --- | --- | --- | --- | --- |
|  | Female | 528 | 271022 | 254760 | 3.781 | 0.0002 | 0.03 |
|  | Male | 436 | 194108 | 210370 |  |  |  |
|  | Combined | 964 | 465130 | 465130 |  |  |  |
| **Visuospatial/ constructional** |  |  |  |  | -4.143 | <0.001 | <0.001 |
|  | Female |  | 236977.5 | 254760 |  |  |  |
|  | Male |  | 228152.5 | 210370 |  |  |  |
|  | Combined |  | 465130 | 465130 |  |  |  |
| **Immediate Memory** |  |  |  |  | 5.884 | <0.001 | <0.001 |
|  | Female |  | 280011.5 | 254760 |  |  |  |
|  | Male |  | 185118.5 | 210370 |  |  |  |
|  | Combined |  | 465130 | 465130 |  |  |  |
| **Delayed Memory** |  |  |  |  |  |  |  |
|  | Female |  | 271768.5 | 254760 | 3.963 | 0.0001 | 0.03 |
|  | Male |  | 193361.5 | 210370 |  |  |  |
|  | Combined |  | 465130 | 465130 |  |  |  |
| **Language** |  |  |  |  | 8.528 | <0.001 | <0.001 |
|  | Female |  | 291368 | 254760 |  |  |  |
|  | Male |  | 173762 | 210370 |  |  |  |
|  | Combined |  | 465130 | 465130 |  |  |  |
| **Attention** |  |  |  |  | 1.063 | 0.288 | 0.8 |
|  | Female |  | 259325.5 | 254760 |  |  |  |
|  | Male |  | 205804.5 | 210370 |  |  |  |
|  | Combined |  | 465130 | 465130 |  |  |  |

**Supplemental Table 5.** Effect size attenuation/reduction due to the inclusion of AD-dementia risk factors was computed as β variable – β (reference). Confidence intervals (CIs), CI attenuation/reduction was computed as CI – CI reference.

|  | **Total Scale** | | **Visuospatial** | | **Immediate Memory** | | **Delayed Memory** | | | **Language** | | | **Attention** | |
| --- | --- | --- | --- | --- | --- | --- | --- | --- | --- | --- | --- | --- | --- | --- |
| Variable | β (95% CI) | β (95% CI) attenuation | β (95% CI) | β (95% CI) attenuation | β (95% CI) | β (95% CI) attenuation | | β (95% CI) | β (95% CI) attenuation | | β (95% CI) | β (95% CI) attenuation | β (95% CI) | β (95% CI) attenuation |
| Reference: Sex (male) | -3.6  (-5.3, -1.9) |  | 4.4  (2.4, 6.4) |  | -5.7  (-7.5, -3.8) |  | | -3.4  (-4.9, -1.9) |  | | -7.0  (-8.6, -5.4) |  | -0.8  (-2.8, 1.2) |  |
| Ethnicity  (white) | -3.7  (-5.5, -2.0) | -0.1  (-0.2, -0.1) | 4.6  (2.6, 6.6) | 0.2  (0.2, 0.2) | -5.3  (-7.8, -4.1) | 0.4  (-0.3, -0.3) | | -3.6  (-5.2, -2.1) | -0.2  (-0.3, -0.2) | | -7.1  (-8.7, -5.5) | -0.1  (-0.1, -0.1) | -0.8  (-2.9, 1.2) | 0  (-0.1, 0) |
| Years of education | -4.2  (-6.0, -2.3) | -0.6  (-0.7, -0.4) | 4.2  (2.1, 6.3) | -0.2  (0.3, -0.1) | -3.9  (-5.7, -4.1) | 1.8  (1.8, -0.9) | | -2.4  (-3.9, -0.9) | 1.0  (1.0, 1.0) | | -6.9  (-8.5, -5.2) | 0.1  (0.1, 0.2) | -1.0  (-3.1, 1.2) | -0.2  (-0.3, 0) |
| Occupation | -6.1  (-7.9, -4.3) | -2.5  (-2.6, -2.4) | 3.7  (1.5, 6.0) | -0.7  (-0.9, -0.4) | -6.2  (-8.3, -4.3) | -0.5  (-0.8, -0.5) | | -3.5  (-5.1, -2.0) | -0.1  (-0.2, -0.1) | | -7.3  (-9.0, -5.7) | -0.3  (-0.4, -0.3) | -1.2  (-3.4, 1.1) | -0.4  (-0.6, -0.1) |
| Household Income | -6.4  (-8.3, -4.6) | -2.8  (-3.0, -2.7) | 2.7  (0.5, 4.9) | -1.7  (-1.9, -1.5) | -7.6  (-9.6, -5.6) | -1.9  (-2.1, -1.8) | | -4.8  (-6.5, -3.2) | -1.4  (-1.6, -1.3) | | -8.4  (-10.1, -6.7) | -1.4  (-1.5, -1.3) | -3.4  (-5.6, -1.2) | -2.6  (-2.8, -2.4) |
| Personal income | -4.1  (-5.9, -2.4) | -0.5  (-0.6, -0.5) | 2.2  (0.04, 4.4) | -2.2  (-2.4, -2.0) | -7.8  (-9.8, -5.7) | -2.1  (-2.3, -1.9) | | -5.0  (-6.6, -3.3) | -1.6  (-1.7, -1.4) | | -8.6  (-10.3, 6.8) | -1.6  (-1.7, -1.4) | -3.6  (-5.8, 1.4) | -2.8  (-3.0, 0.2) |
| Married | -3.3  (-5.1, -1.6) | 0.3  (0.2, 0.3) | 4.1  (2.0, 6.1) | -0.3  (-0.4, -0.3) | -6.2  (-8.0, -4.3) | -0.5  (-0.5, -0.5) | | -3.7  (-5.3, -2.2) | -0.3  (-0.4, -0.3) | | -7.5  (-9.2, -5.9) | -0.5  (-0.6, -0.5) | -1.0  (-3.0, 1.1) | -0.2  (-0.2, -0.1) |
| BMI | -3.0  (-4.8, -1.3) | 0.6  (0.5, 0.6) | 5.0  (3.0, 7.1) | 0.6  (0.6, 0.7) | -5.7  (-7.6, -3.8) | 0  (-0.1, 0) | | -3.5  (-5.1, -1.9) | -0.1  (-0.2, 0) | | -7.1  (-8.8, -5.4) | -0.1  (-0.2, 0) | -0.3  (-2.3, 1.8) | 0.5  (0.5, 0.6) |
| Hypertension | -3.3  (-5.0, -1.6) | 0.3  (0.3, 0.3) | 4.9  (2.9, 6.9) | 0.5  (0.5, 0.5) | -5.3  (-7.2, -3.4) | 0.4  (-0.3, 0.4) | | -3.5  (-4.7, -1.6) | -0.1  (0.2, 0.3) | | -6.7  (-8.3, -5.0) | 0.3  (-0.3, 0.4) | -0.1  (-2.2, 1.9) | 0.7  (0.6, 0.7) |
| Diabetes | -3.6  (-5.3, -1.8) | 0  (0, 0.1) | 4.6  (2.6, 6.7) | 0.2  (0.2, 0.3) | -5.4  (-7.3, -3.6) | 0.3  (-0.2, -0.2) | | -3.3  (-4.8, -1.8) | 0.1  (0.1, 0.1) | | -6.8  (-8.4, -5.1) | 0.2  (0.2, 0.3) | -0.5  (-2.5, 1.5) | 0.3  (-0.3, 0.3) |
| Stroke | -3.6  (-5.3, -1.8) | 0  (0, 0.1) | 4.4  (2.4, 6.4) | 0  (0, 0) | -5.7  (-7.5, -3.8) | 0  (0, 0) | | -3.4  (-4.9, -1.8) | 0  (0, 0.1) | | -7.0  (-8.6, -5.4) | 0  (0, 0) | -0.8  (-2.8, 1.2) | 0  (0, 0) |
| CVD | -3.6  (-5.3, -1.8) | 0  (0, 0.1) | 4.5  (2.5, 6.5) | 0.1  (0.1, 0.1) | -5.7  (-7.6, -3.9) | 0  (-0.1, -0.1) | | -3.3  (-4.9, -1.8) | 0  (0, 0.1) | | -7.0  (-8.7, -5.4) | 0  (-0.1, 0) | -0.7  (-2.8, 1.3) | 0.1  (0, -0.1) |
| Alcohol | -4.9  (-6.7, -3.1) | -1.3  (-1.4, -1.2) | 3.7  (1.7, 5.8) | -0.7  (-0.7, -0.6) | -6.8  (-8.8, -4.8) | -1.1  (-1.3, -1.0) | | -3.9  (-5.5, -2.2) | -0.5  (-0.6, -0.3) | | -8.2  (-9.9, -6.4) | -1.2  (-1.3, -1.0) | -2.2  (-4.4, 0.003) | -1.4  (-1.6, -1.2) |
| Fruit | -3.8  (-5.6, -1.9) | -0.2  (-0.3, 0) | 4.4  (2.3, 6.5) | 0  (-0.1, 0.1) | -5.8  (-7.8, -3.9) | -0.1  (-0.3, -0.1) | | -3.0  (-4.7, -1.4) | 0.4  (0.2, 0.5) | | -7.2  (-8.9, -5.4) | -0.2  (-0.3, 0) | -1.4  (-3.5, 0.8) | -0.6  (-0.7, -0.4) |
| Vegetables | -3.9  (-5.8, -2.1) | -0.3  (-0.5, -0.2) | 4.5  (2.4, 6.6) | 0.1  (0, 0.2) | -6.0  (-8.0, -4.0) | -0.3  (-0.5, -0.2) | | -3.0  (-4.6, -1.4) | 0.4  (0.3, 0.5) | | -7.3  (-9.1, -5.6) | -0.3  (-0.5, -0.2) | -1.7  (-3.9, 0.5) | -0.9  (-1.1, -0.7) |
| Smoking | -3.8  (-5.6, -2.1) | -0.2  (0.3, -0.2) | 4.2  (2.2, 6.2) | -0.2  (-0.2, -0.2) | -5.8  (-7.7, -4.0) | -0.1  (-0.2, -0.2) | | -3.4  (-5.0, -1.9) | 0  (-0.1, 0) | | -7.2  (-8.9, -5.6) | -0.2  (-0.3, -0.2) | -0.9  (-3.0, 1.1) | -0.1  (-0.2, -0.1) |
| Physical Activity | -4.5  (-6.3, -2.7) | -0.9  (-1.0, -0.8) | 4.1  (2.0, 6.2) | -0.3  (-0.4, -0.2) | -6.4  (-8.3, -4.5) | -0.7  (-0.8, -0.7) | | -4.1  (-6.7, -2.5) | -0.7  (-1.8, -0.6) | | -7.4  (-9.1, -5.7) | -0.4  (-0.5, -0.3) | -1.8  (-3.9, 0.3) | -1.0  (-1.1, -0.9) |
| Depression | -3.6  (-5.3, -1.9) | 0  (0, 0) | 4.5  (2.5, 6.5) | 0.1  (0.1, 0.1) | -5.7  (-7.5, -3.8) | 0  (0, 0) | | -3.3  (-4.8, -1.8) | 0.1  (0.1, 0.1) | | -7.0  (-8.6, -5.4) | 0  (0, 0) | -0.9  (-2.9, 1.2) | -0.1  (0,0,-0.1) |

**Supplemental Table 6.** Summarises the Cohen’s U3 values for the RBANS indices, indicating the proportion of females who scored above the age-adjusted mean of males at baseline.

| RBANS domain | Cohen’s U3 value | Interpretation |
| --- | --- | --- |
| ***Total Scale*** | 0.4 | Approximately 40% of females score above the adjusted mean of males. |
| ***Visuospatial/constructional*** | 0.6 | Approximately 60% of females score above the adjusted mean of males. |
| ***Immediate Memory*** | 0.35 | Approximately 35% of females score above the adjusted mean of males. |
| ***Delayed Memory*** | 0.4 | Approximately 40% of females score above the adjusted mean of males. |
| ***Language*** | 0.3 | Approximately 30% of females score above the adjusted mean of males. |
| ***Attention*** | 0.5 | There is no difference between the men and women. |

**Supplemental Table 7.** Multiple regression analyses with progressive adjustment for confounders to assess the association between sex and cognitive performance in the RBANS indices at baseline in the CPRO-MS (unimputed complete case analysis).

| **Model No.** | **N** | *Difference in mean scores*  **men compared to women (ref.) (95% CI)** | **p** | *Difference in mean scores*  **men compared to women (ref.) (95% CI)** | | **p** |  |  |  |
| --- | --- | --- | --- | --- | --- | --- | --- | --- | --- |
|  |  |  |  |  |  |  |  | |  |
|  |  | **Total Scale** | | | **Visuospatial/Constructional** | | |  | |
| **1** | 915 | -3.7 (-5.4, -2.0) | <0.001 | 4.6 (2.6, 6.6) | | <0.001 |  | |  |
| **2** | 543 | -4.3 (-6.4, -2.3) | <0.001 | 3.8 (1.3, 6.4) | | 0.003 |  | |  |
| **3** | 473 | -4.4 (-6.5, -2.2) | <0.001 | 3.4 (0.7, 6.1) | | 0.01 |  | |  |
|  |  | **Immediate Memory** | | | **Delayed Memory** | | |  | |
| **1** | 915 | -5.9 (-7.8, -4.1) | <0.001 | -3.6 (-5.1, -2.0) | | <0.001 |  | |  |
| **2** | 543 | -4.8 (-7.0, -2.6) | <0.001 | -2.9 (-4.8, -1.1) | | 0.002 |  | |  |
| **3** | 473 | -4.9 (-7.3, -2.6) | <0.001 | -2.4 (-4.4, -0.4) | | 0.02 |  | |  |
|  |  | **Language** | | | **Attention** | | |  | |
| **1** | 915 | -7.1 (-8.7, -5.5) | <0.001 | -0.8 (-2.9, 1.2) | | 0.4 |  | |  |
| **2** | 543 | -7.9 (-9.8, -5.9) | <0.001 | -2.6 (-5.3, 0.07) | | 0.06 |  | |  |
| **3** | 473 | -8.0 (-10.1, -5.9) | <0.001 | -2.6 (-5.5, 0.3) | | 0.08 |  | |  |

Model 1: ethnicity, Model 1 + 2: years of education, personal annual income, occupation & marital status, and Model 1, 2 + 3: CVD, hypertension, diabetes, stroke, BMI, smoking status, alcohol intake, food & vegetable consumption, physical activity & depression. Significance level was set at 0.01.

**Supplemental Table 8.** The effect of time on cognitive performance in the RBANS, N=691.

| **RBANS: Cognitive performance, N= 691** | **change in mean scores per year β (95% CI)** | **p** |
| --- | --- | --- |
| Total scale | 1.2 (0.7, 1.8) | <0.001 |
| Visuospatial/constructional | -2.8 (-3.7, -2.0) | <0.001 |
| Immediate memory | 2.8 (2.1, 3.5) | <0.001 |
| Delayed memory | 2.0 (1.5, 2.5) | <0.001 |
| Language | -0.03 (-0.6, 0.7) | 0.9 |
| Attention | 2.2 (1.5, 2.9) | <0.001 |

Significance level was set at 0.01.

**Supplemental Table 9.** Linear mixed model analyses to assess the interaction between sex and time on the mean change in cognitive trajectories of the RBANS per year, adjusted for Models 1 & 2. imputed model, N=691.

| **β (95% CI)** | **p** |
| --- | --- |
| **Total Scale** | |
| Model 1 main effect: -1.1 (-2.8, 0.6) | 0.2 |
| Model 1 interaction term: -1.3 (-2.1, -0.5) | 0.002 |
| Model 2 main effect: -2.3 (-4.0, -0.6) | 0.009 |
| Model 2 interaction term: -1.3 (-2.1, -0.4) | 0.003 |
| **Visuospatial/constructional Index** | |
| Model 1 main effect: 5.2 (3.2, 7.1) | <0.001 |
| Model 1 interaction term: -1.6 (-2.8, -0.4) | 0.009 |
| Model 2 main effect: 5.2 (3.2, 7.2) | <0.001 |
| Model 2 interaction term: -1.6 (-2.8, -0.4) | 0.009 |
| **Immediate Memory** | |
| Model 1 main effect: -3.2 (-4.9, -1.5) | <0.001 |
| Model 1 interaction term: -1.8 (-2.9, -0.8) | 0.001 |
| Model 2 main effect: -4.1 (-5.8, -2.4) | p<0.001 |
| Model 2 interaction term: -1.8 (-2.9, -0.8) | 0.001 |
| **Delayed Memory** | |
| Model 1 main effect: -1.2 (-2.5, 0.2) | 0.09 |
| Model 1 interaction term: -1.2 (-2.0, -0.4) | 0.003 |
| Model 2 main effect: -1.5 (-2.9, -0.05) | 0.04 |
| Model 2 interaction term: -1.2 (-2.0, -0.4) | 0.003 |
| **Language** | |
| Model 1 main effect: -4.0 (-5.5, -2.4) | <0.001 |
| Model 1 interaction term: 1.1 (0.02, 2.1) | 0.05 |
| Model 2 main effect: -5.2 (-6.8, -3.7) | <0.001 |
| Model 2 interaction term: 1.1 (0.03, 2.1) | 0.04 |
| **Attention** | |
| Model 1 main effect: -0.1 (-2.2, 2.1) | 1 |
| Model 1 interaction term: -0.5 (-1.5, 0.4) | 0.3 |
| Model 2 main effect: -1.8 (-4.0, 0.5) | 0.1 |
| Model 2 interaction term: -0.5 (-1.5, 0.4) | 0.3 |

Model 1: ethnicity, Model 1 + 2: years of education, personal annual income, occupation & marital status, Significance level was set at 0.01.

**Supplemental Table 10.**  Linear mixed model analyses to assess the interaction between sex and time on the mean change in cognitive trajectories of the RBANS per year in the CPRO-MS, (unimputed complete case analysis, **N= 370)**.

|  | **Total Scale** | | **Visuospatial/Constructional** | |
| --- | --- | --- | --- | --- |
| **Main Effect: Sex^1^** | -4.9 (-7.2, -2.6) | <0.001 | 3.4 (0.8, 6.0) | 0.01 |
| **Interaction between sex & time^2^** | -0.4 (-1.5, 0.7) | 0.3 | -1.7 (-3.4, -0.1) | 0.04 |
| **Immediate Memory** | | | **Delayed Memory** | |
| **Main Effect: Sex^1^** | -6.0 (-8.3, -3.6) | <0.001 | -1.8 (-3.8, 0.2) | 0.08 |
| **Interaction between sex & time^2^** | -0.5 (-2.0, 1.0) | 0.5 | -1.2 (-2.4, 0.01) | 0.05 |
| **Language** | | | **Attention** | |
| **Main Effect: Sex^1^** | -7.3 (-9.5, -5.2) | <0.001 | -4.6 (-7.6, -1.7) | 0.002 |
| **Interaction between sex & time^2^** | 2.3 (0.7, 3.8) | 0.004 | -0.3 (-1.7, 1.1) | 0.7 |

Adjusted for ethnicity, years of education, personal annual income, occupation, marital status, CVD, hypertension, diabetes, stroke, BMI, smoking status, alcohol consumption, physical activity & depression. ^1^Difference in mean scores, men compared to women. ^2^Change in mean scores per year in men compared to women (practice effects). Significance level was set at 0.01.

**Supplemental Table 11.** The interaction of sex and time on cognitive trajectories of the RBANS scores per year for participants in the CPRO-MS, (waves 1-6, imputed model), N=691.

| **Model 3** | **β (95% CI)** | **p** |
| --- | --- | --- |
|  | **Total Scale** | |
| **Main effect: Sex^1^** | -2.6 (-4.4, -0.7) | 0.006 |
| **Interaction between sex & time^2^** | -1.3 (-2.1, -0.5) | 0.002 |
| **Visuospatial/Constructional Index** | | |
| **Main effect: Sex^1^** | 5.2 (3.1, 7.3) | <0.001 |
| **Interaction between sex & time^2^** | -1.6 (-2.8, -0.4) | 0.01 |
| **Immediate Memory** | | |
| **Main effect: Sex^1^** | -4.4 (-6.2, -2.6) | <0.001 |
| **Interaction between sex & time^2^** | -1.9 (-3.0, -0.8) | <0.001 |
| **Delayed Memory** | | |
| **Main effect: Sex^1^** | -1.5 (-3.0, -0.04) | 0.04 |
| **Interaction between sex & time^2^** | -1.3 (-2.1, -0.5) | 0.002 |
| **Language** | | |
| **Main effect: Sex^1^** | -5.5 (-7.2, -3.8) | <0.001 |
| **Interaction between sex & time^2^** | 1.1 (0.05, 2.1) | 0.04 |
| **Attention** | | |
| **Main effect: Sex^1^** | -2.1 (-4.4, 0.3) | 0.09 |
| **Interaction between sex & time^2^** | -0.5 (-1.4, 0.5) | 0.3 |

Adjusted for ethnicity, years of education, personal annual income, occupation, marital status, CVD, hypertension, diabetes, stroke, BMI, smoking status, alcohol consumption, physical activity & depression. ^1^Difference in mean scores, men compared to women. ^2^Change in mean scores per year in men compared to women (practice effects). Significance level was set at 0.01.

**Supplemental Table 12.** Age-stratified linear regression analyses to assess the association between sex and time on and cognitive performance in the RBANS in ‘younger’ verses ‘older’ participants in the CPRO-MS, N=691.

| **Men compared to women (ref)** | **Change in mean scores per year**  **β (95% CI)** | **p** | **Change in mean scores per year**  **β (95% CI)** | **p** |
| --- | --- | --- | --- | --- |
| **Model** | **Total scale** | | **Visuospatial/Constructional** | |
| 1. Interaction between sex and time (60-68 years) N=351 | -0.3 (-1.7, 1.1) | 0.7 | -1.1 (-3.6, 1.5) | 0.4 |
| 2. Interaction between sex and time (69-85 years) N= 340 | -1.9 (-3.5, -0.3) | 0.02 | -2.6 (-4.7, -0.6) | 0.01 |
| **Immediate Memory** | | | **Delayed Memory** | |
| **1** | -0.8 (-2.8, 1.1) | 0.4 | -0.9 (-2.3, 0.6) | 0.3 |
| **2** | -3.2 (-5.2, -1.2) | 0.002 | -1.1 (-2.7, 0.4) | 0.2 |
| **Language** | | | **Attention** | |
| **1** | 2.6 (0.8, 4.3) | 0.004 | -0.1 (-2.2, 2.0) | 0.9 |
| **2** | 1.3 (-0.6, 3.2) | 0.2 | -0.8 (-2.7, 1.0) | 0.4 |

Significance level was set at 0.01.

**Supplemental Table 13.** *APOE* ε4 stratified linear regression analyses to assess the association between sex and time on and cognitive performance in the RBANS in ‘carriers’ verses ‘non-carriers’ of the allele in the CPRO-MS, N=691.

| **Men compared to women (ref)** | **Change in mean scores per year**  **β (95% CI)** | **p** | **Change in mean scores per year β (95% CI)** | | **p** |  |
| --- | --- | --- | --- | --- | --- | --- |
| **Model** | **Total scale** | | **Visuospatial/Constructional** | | |  |
| 1. Interaction between sex and time (*APOE* ε4 Carrier) N=164 | -2.6 (-4.2, -0.9) | 0.002 | -3.4 (-6.3, -0.3) | | 0.03 |  |
| 2. Interaction between sex and time (Non- Carrier) N=527 | -0.7 (-2.1, 0.6) | 0.3 | -2.9 (-5.7, -0.06) | | 0.05 |  |
| **Immediate Memory** | | | | **Delayed Memory** | | |
| 1 | -1.8 (-4.2, 0.5) | 0.1 | -2.5 (-4.1, -0.9) | | 0.002 |  |
| 2 | -2.1 (-3.8, -0.4) | 0.02 | -0.7 (-2.0, 0.6) | | 0.3 |  |
| **Language** | | | | **Attention** | | |
| 1 | 1.2 (-0.7, 3.1) | 0.2 | -2.9 (-4.9, -0.8) | | 0.006 |  |
| 2 | 2.1 (0.5, 3.7) | 0.01 | 0.3 (-1.4, 2.1) | | 0.7 |  |

Significance level was set at 0.01.

**Supplemental Table 14.** *APOE* ε4 stratified linear regression analyses to assess the association between sex and time on and cognitive performance in the RBANS in ‘carriers’ verses ‘non-carriers’ (excluding carriers of the ε2 allele) of the ε4 allele in the CPRO-MS, N=668.

| **Sample excluding E2** | **Change in mean scores per year**  **β (95% CI)** | **p** | **Table from manuscript including E2** | **Change in mean scores per year**  **β (95% CI)** | **p** |
| --- | --- | --- | --- | --- | --- |
| **Model No** | **Total scale** | | | | |
| 1. (*APOE* ε4 Carrier) N=142 | -1.3 (-2.7, 0.2) | 0.08 | 1. (*APOE* ε4 Carrier) N=164 | -2.6 (-4.2, -0.9) | 0.002 |
| 2. (Non-Carrier)  N=526 | -0.7 (-1.7, 0.3) | 0.1 | 2. (Non-Carrier) N=527 | -0.7 (-2.1, 0.6) | 0.3 |
| **Immediate Memory** | | | | | |
| 1. (*APOE* ε4 Carrier) N=142 | -1.1 (-2.4, 0.2) | 0.1 | 1. (*APOE* ε4 Carrier) N=164 | -1.8 (-4.2, 0.5) | 0.1 |
| 2. (Non-Carrier)  N=526 | -0.6 (-2.5, 1.3) | 0.5 | 2. (Non-Carrier) N=527 | -2.1 (-3.8, -0.4) | 0.02 |
| **Language** | | | | | |
| 1. (*APOE* ε4 Carrier) N=142 | 1.7 (0.4, 2.9) | 0.01 | 1. (*APOE* ε4 Carrier) N=164 | 1.2 (-0.7, 3.1) | 0.2 |
| 2. (Non-Carrier)  N=526 | 2.1 (0.3, 3.9) | 0.02 | 2. (Non-Carrier) N=527 | 2.1 (0.5, 3.7) | 0.01 |
|  | **Visuospatial/Constructional** | | | | |
| 1. (*APOE* ε4 Carrier) N=142 | -1.3 (-2.8, 0.1) | 0.07 | 1. (*APOE* ε4 Carrier) N=164 | -3.4 (-6.3, -0.3) | 0.03 |
| 2. (Non-Carrier)  N=526 | -1.2 (-3.4, 1.0) | 0.3 | 2. (Non-Carrier) N=527 | -2.9 (-5.7, -0.06) | 0.05 |
|  | **Delayed Memory** | | | | |
| 1. (*APOE* ε4 Carrier) N=142 | -0.5 (-1.4, 0.5) | 0.3 | 1. (*APOE* ε4 Carrier)  N=164 | -2.5 (-4.1, -0.9) | 0.002 |
| 2. (Non-Carrier)  N=526 | -2.0 (-3.4, -0.6) | 0.004 | 2. (Non-Carrier) N=527 | -0.7 (-2.0, 0.6) | 0.3 |
|  | **Attention** | | | | |
| 1. (*APOE* ε4 Carrier) N=142 | -0.3 (-1.4, 0.8) | 0.006 | 1. (*APOE* ε4 Carrier) N=164 | -2.9 (-4.9, -0.8) | 0.006 |
| 2. (Non-Carrier)  N=526 | -1.5 (-3.2, 0.1) | 0.7 | 2. (Non-Carrier) N=527 | 0.3 (-1.4, 2.1) | 0.7 |
